# Associations between Family Functioning and Maternal Behavior on Default Mode Network Connectivity in School-Age Children

**DOI:** 10.3390/ijerph19106055

**Published:** 2022-05-16

**Authors:** Keila Rebello, Luciana Monteiro Moura, Ana Paula Arantes Bueno, Felipe Almeida Picon, Pedro Mario Pan, Ary Gadelha, Euripedes Constatino Miguel, Rodrigo Affonseca Bressan, Luis Augusto Rohde, João Ricardo Sato

**Affiliations:** 1 Center of Mathematics, Computing and Cognition, Universidade Federal do ABC, Santo André 09210-580, Brazil; keila.rebello@ufabc.edu.br (K.R.); lummoura@gmail.com (L.M.M.); apbueno74@gmail.com (A.P.A.B.); 2 Interdisciplinary Lab for Clinical Neurosciences, Universidade Federal de São Paulo, São Paulo 04021-001, Brazil; pedro.pan@unifesp.br (P.M.P.); aryararipe@gmail.com (A.G.); rodrigoabressan@gmail.com (R.A.B.); 3 National Institute of Developmental Psychiatry for Children and Adolescents (CNPq), São Paulo 01060-970, Brazil; felipepicon@gmail.com (F.A.P.); ecmiguel@usp.br (E.C.M.); larohde@gmail.com (L.A.R.); 4 ADHD Outpatient Program & Developmental Psychiatry Program, Hospital de Clínicas de Porto Alegre, Federal University of Rio Grande do Sul, Porto Alegre 90010-150, Brazil; 5 Department of Psychiatry, School of Medicine, University of São Paulo, São Paulo 05508-070, Brazil

**Keywords:** default-mode network, resting State, family environment, parental practices childhood, adolescence

## Abstract

**Background:** Most early children’s experiences will occur in a family context; therefore, the quality of this environment is critical for development outcomes. Not many studies have assessed the correlations between brain functional connectivity (FC) in important areas such as the default mode network (DMN) and the quality of parent-child relationships in school-age children and early adolescence. The quality of family relationships and maternal behavior have been suggested to modulate DMN FC once they act as external regulators of children’s affect and behavior. **Objective:** We aimed to test the associations between the quality of family environment/maternal behavior and FC within the DMN of school-age children. **Method:** Resting-state, functional magnetic resonance imaging data, were collected from 615 children (6–12 age range) enrolled in the Brazilian High-Risk Cohort (HRC) study. We assessed DMN intra-connectivity between the medial prefrontal cortex (mPFC), posterior cingulate cortex (PCC), and inferior parietal lobule (IPL-bilateral) regions. The family functioning was assessed by levels of family cohesiveness and conflict and by maternal behavior styles such as maternal responsiveness, maternal stimulus to the child’s autonomy, and maternal overprotection. The family environment was assessed with the Family Environment Scale (FES), and maternal behavior was assessed by the mother’s self-report. **Results:** We found that the quality of the family environment was correlated with intra-DMN FC. The more conflicting the family environment was, the greater the FC between the mPFC-left IPL (lIPL), while a more cohesive family functioning was negatively correlated with FC between the PCC-lIPL. On the other hand, when moderated by a positive maternal behavior, cohesive family functioning was associated with increased FC in both regions of the DMN (mPFC-lIPL and PCC-lIPL). **Conclusions:** Our results highlight that the quality of the family environment might be associated with differences in the intrinsic DMN FC.

## 1. Introduction

The protracted maturation of the human brain is largely dependent on early postnatal experiences [1], for a review of [2]. Most of these early experiences will occur in a family context, and therefore, the quality of this environment is critical for the outcomes of children’s developmental process [3,4]. Some studies have explored the consequences of different socio-economic backgrounds [5,6,7], effects of poverty [8,9,10], and the influences of parental income and education [6,11,12] on brain networks to illustrate some effects of family experiences on children’s brain development.

Brain regions associated with the development of self-regulatory abilities, such as those involved in self-referential activity [13,14,15], social cognition [16,17], and empathy [18] are of special interest as they have been extensively related to the default mode network (DMN) functions [19,20].

The DMN is a resting-state network composed of the medial prefrontal cortex (mPFC), posterior cingulate cortex (PCC), and bilateral inferior parietal lobule (IPL). It is thought to support self-related processes [13,19,21,22]. Although all regions share functions in these self-related processes, many specificities have been suggested (e.g., mPFC’s role in mentalizing self, evaluation, and perspective-taking [23,24]; PCC is thought to regulate the balance between internal and external focus [25]; IPL seems to be relevant for self-other discrimination [21,26] and judgment of social closeness between people [27,28].

Altered DMN functional connectivity (FC) related to deficits in those mentioned cognitive functions has been described in a wide range of psychiatric and neurodevelopmental disorders, such as autism [29,30,31], attention-deficit/hyperactivity disorder [32,33,34], mood disorders [35,36], schizophrenia [37,38], and bipolar disorder [39,40]. Only a few studies have assessed the association between the quality of parent-child relationship and DMN regions [5,41,42,43].

The effect of parent-child interactions might be important considering that they potentially work as external regulators of children’s affect and behavior [44,45]. There is evidence suggesting that the quality of parent-child interactions mediate differences in the DMN neural FC. Negative interaction, characterized by lower family cohesion and a greater family conflict, has been associated with the increased activation of PCC and the ventrolateral prefrontal cortex (vlPFC), regions considered to be the neural substrates of increased risk behaviors in adolescents [42]. Lower parental responsiveness was associated with increased FC between mPFC and amygdala in children aged 4–10 years old [43]. Likewise, exposure to higher parental conflict (verbal conflict) and increased FC between PCC and the anterior portion of mPFC and between PCC and amygdala were found in infants aged 6–12 months. These infants also exhibited higher negative emotional reactivity [41]. On the other hand, positive family relationships characterized by high levels of cohesion, communication, and warmth [9,11] have distinct neural development outcomes. A more mature brain FC was described by Degeilh et al. [5], and a better quality of maternal behavior was predictive of a stronger negative FC between DMN regions (anterior cingulate cortex and ventral mPFC) and salience network in children from birth until nine years later. They described higher levels of mind-mindedness (attention and sensibility to child’s ongoing mental state) and autonomy support (encouragement of child independence for problem-solving and a focus on the child’s own decisions) as main contributors to self-regulatory abilities attributed to DMN (as their possible neural substrate).

Thus, these studies show that the quality of family environment (negative or positive) and the quality of parental caregiving are associated with variations in DMN regions, specifically the PCC and the mPFC portions. The FC of these two main regions with the bilateral IPL has been investigated less, despite its role in perspective-taking and the judgment of social closeness between people [27] and social perception [46], which are important self-referential processes attributed to the DMN.

In this study, we aimed to test the associations between the quality of family environment/maternal behavior and FC within the DMN of school-age children. This is a hypothesis-driven study focused on testing two-hypothesis: (1) if there is an association between children‘s FC of PCC and mPFC regions, and its connections with bilateral IPL [left inferior parietal lobule (lIPL) and the right inferior parietal lobule (rIPL)] and the quality of the family environment; and (2) if there is an association between these two DMN regions and the the quality of family environment when mediated by maternal behavior. We hypothesized that higher conflict in the family functioning would be associated with increased FC between mPFC-IPL [41]. A lower cohesive functioning was expected to be associated with increased FC in the PCC-IPL regions [5], and maternal behavior associations with cohesive functioning would increase the FC between both mPFC-IPL and PCC-IPL.

## 2. Materials and Methods

### 2.1. Participants

**Figure 1:**
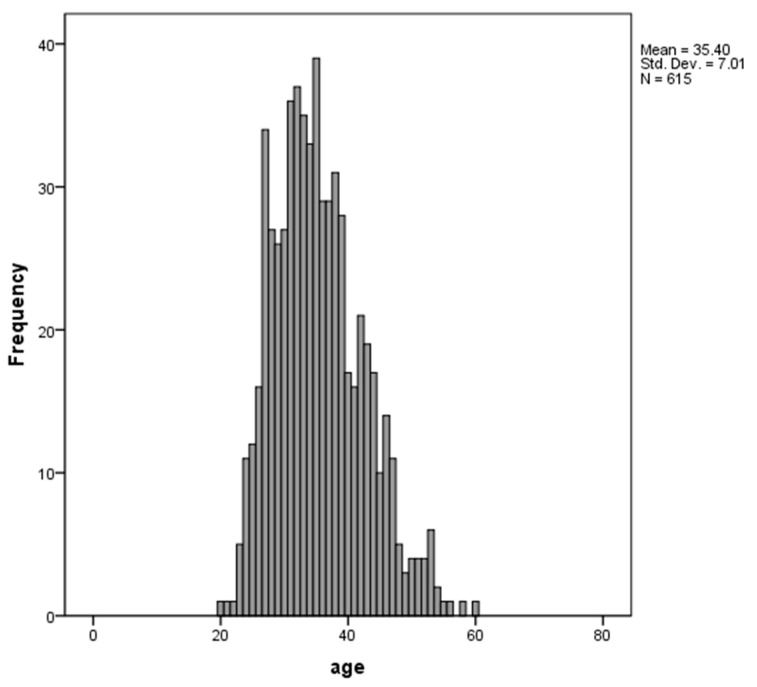
Histogram and descriptive statistics of participants age (in months).

We included 615 school-age children (6-to-12-years-old, see Figure 1) participating in the “Brazilian High-Risk Cohort (HRC) Study for Psychiatric Disorders in Childhood”. The HRC is a population-based sample from 57 Brazilian public schools (35 schools in the city of São Paulo and 22 schools in Porto Alegre). Further details on this cohort can be found elsewhere [47]. Written informed consent was obtained for each participant, and all experimental procedures were in accordance with local regulations and approved by local ethics committees.

### 2.2. Behavioral Assessments

The family environment was assessed using the Family Environment Scale (FES); [48,49]. The FES assesses interpersonal relationships and the social environment from the point of view of a family member by quantifying three domains: interpersonal relationships, directions of personal growth, and basic organization. Interpersonal family relationship was evaluated through the subscales of conflict and cohesion. The parents answered all scales during home interviews (biological mothers were 87.6% of the informants). The items from both subscales were loaded into a general latent factor (general interpersonal family functioning), and the residuals from each indicator were loaded into specific factors of cohesion and conflict. This bifactor model gave a good fit to the data (RMSEA = 0.029, CI90% 0.026 to 0.033; CFI = 0.985, TLI 0.98). Further information about the model and fitting is described in Sato et al., (2018). Higher scores reflect a higher quality of the family environment on the cohesion scale, and lower scores show the inverse on the conflict scale.

To investigate the features of parental caregiving, three questions related to maternal behavior were included: (1) maternal responsiveness (mother smiles at the child, speaks friendly, praises child’s actions, tries to understand the child, and helps with problem-solving); (2) maternal stimulation to child’s autonomy (mother gives the child autonomy in choices); (3) maternal overprotection (mother treats the child as a baby, is overprotective, wishes to prevent the child from growing up). These three questions were summarized as a single factor named “maternal behavior”. We acknowledge that this variable is limited in its scope. However, since the participants in this cohort were assessed using several questionnaires regarding a myriad of constructs, unfortunately, it was not possible to obtain very detailed information on maternal caregiving.

Regarding the characterization of this sample, the Brazilian socioeconomic scale ABEP; 2010 version by Almeida and Whickerhauser [50] was used to define the socioeconomic scores (SES) of families. The quantitative SES was calculated for each family, as only one child per family was enrolled in the HRC study.

### 2.3. MRI Acquisition

Resting-state functional neuroimaging (rs-fMRI) data were obtained in two 1.5 T MRI GE scanners (São Paulo city, Brazil: Signa HDX and Porto Alegre: Signa HD) with identical acquisition parameters, as follows: 1080 whole-brain EPI volumes were obtained for each participant (TR = 2000 ms, TE = 30 ms, slice thickness = 4 mm, gap = 0 mm, flip angle = 80°, matrix size = 80 × 80, reconstruction matrix = 128 × 128, 1.875 × 1.875 mm, NEX = 1, slices = 26, total acquisition time of six minutes). Participants were instructed to keep their eyes open and fixate their gaze at a painted target. T1-weighted scans (3D FSPGR sequence) were obtained considering up to 160 axial slices (TR = 10.91 ms, TE = in phase 4.2 ms, thickness = 1.2 mm, flip angle = 15°, matrix size = 256 × 192, FOV = 24 × 18 cm, NEX = 1).

### 2.4. MRI Preprocessing

rs-fMRI image preprocessing was performed with the CONN toolbox v.16.b (https://www.nitrc.org/projects/conn/ accessed on 12 November 2019). In brief, the first four scans were dropped in order to achieve a steady-state condition. Preprocessing steps were performed using a standard pipeline that included: realignment and unwarping, slice-timing correction, structural segmentation [white matter (WM), grey matter (GM), and cerebrospinal fluid (CSF)], spatial normalization resulting in both functional and structural images in the Montreal Neurological Institute (MNI)-space, outlier detection, smoothing, denoising with a simultaneous option for the removal of WM and CSF noise (with five dimensions each), scrubbing, motion regression (12 regressors: six motion parameters + six first-order temporal derivatives), and band-pass filtering (from 0.008 Hz to 0.09 Hz).

The DMN regions of interest (ROIs) were defined as proposed by Fox et al., (2005), with three predefined seed regions (MNI coordinates): mPFC (−1, 47, −4), PCC (−5, −49, 40), and LPC (−45, −67, 36). The mPFC, PCC, and IPL (left IPL and right IPL, lIPL, and rIPL, respectively) were chosen as they represent the core-self DMN regions, each of them presenting specificities on their support to self-referential functions [13,15]. Importantly, they belong to a functional network characterized by maturation heterogeneity between its anterior-posterior regions [51,52,53] which are relevant aspects of our chosen seeds and are critical to interpreting our results.

FC estimates among the ROIs were obtained using Pearson’s correlation coefficient for the preprocessed mean blood oxygen level-dependent (BOLD) signal of each ROI. These correlations were then normalized using the Fisher transform.

### 2.5. Statistical Analyses

For each seed region, associations between FC, each pair of DMN nodes (PCC-lIPL, PCC-rIPL, mPFC-lIPL, mPFC-rIPL, mPFC-PCC), and family functioning (FES conflict and FES cohesion) were tested using the general linear model (GLM), considering the correlation value between each pair of DMN nodes as the dependent variable, FES scales as the independent variables, and age, gender, and site of MRI acquisition as covariates. SES was tested as a covariate. In addition, we included FES and its interactions with the maternal behavior factors as variables of interest. The Type I Error was set at 5%. Since the sample is large, the GLM assumption on residual normality is not necessary due to the Hajek–Sidak central limit theorem.

To facilitate the GLM analysis, a Principal Component Analysis (PCA) was carried out to reduce the three questions related to the maternal behavior factors, to facilitate the GLM analysis and the quality of the child’s family environment when mediated by the maternal behavior (2).

## 3. Results

The demographic characteristics of the participants are given in Table 1 and Figure 1. The mean age of the participants was 8.68 years old (standard deviation [SD] 1.79), and 55.6% were male (*n* = 342). The Mean Intelligence Coefficient was 102.5 (SD 16.60; median 100). Considering the scores of family functioning, family FES cohesion was slightly higher than FES conflict scores.

Regarding the questionnaires, the gender of the respondents (parents), 90.4% (556 participants) were female and 9.6% (N = 59) were male. The mean family income was 2865.36 (s.d. = 2329.61) Brazilian currency (BRL). Since the birth of the child, 52.8% of the respondents reported that the financial condition of the families improved, and 15.2% reported a deterioration. The parent that spent more time with the child was the mother (94%). and 99% of them were still in contact with the respective child (0.7% missing and 0.3% passed away), and 53.2% of them were still living with the biological father of the child (14.3% were divorced). Regarding the skin color of the mother, 57.6% were white, 16.1% black, 25.4% pardo, and the remaining were others (yellow, indigenous, or do not know). In terms of the mothers‘ educational status, 2.4% never went to school, 14.8% completed elementary school, 33.48% high school, and 3.4% went to college. Moreover, 45.5% of the mothers had a permanent job, and 6.5% were unemployed.

The variance explained for maternal behavior questions were 43.27%, 33.13%, and 23.59% for first, second, and third PCA, respectively. The extracted commonalities for questions 1, 2, and 3 were 64.2%, 44.8%, and 20.8%, respectively. The coefficient score for the first PCA component was 0.617, 0.516, and 0.351 for each question, respectively. From now on, we will refer to “maternal behavior”, the variable of the first PCA component (i.e., the higher variance).

According to Table 2, a significant and positive interaction effect (GLM beta coefficient) was found between mPFC-lIPL and FES conflict (*p* = 0.02), but no significant interactions were found between FES cohesion and any pair of regions. Interestingly, maternal behavior interaction was significant and positive for the PCC-lIPL pair (*p* = 0.02). No other interactions were statistically significant.

## 4. Discussion

Here we have investigated the associations between the quality of family functioning and intrinsic DMN FC in a large sample of school-aged children. Our results suggest that differences in DMN FC may occur according to the variations in the quality of family relationships. To the best of our knowledge, there is no other study relating family environment influences to intra-DMN FC, except for those previous investigations exclusively focused on PCC-mPFC connections.

An increased FC between mPFC (the most anterior DMN region) and the lIPL was previously associated with poorer self-reflection to stronger cognitive demands in schizophrenic patients [54], while in healthy individuals, the lIPL activations result from an increase in attentional demand (which engage both frontal and parietal regions) during behavioral sequence execution [55]. We argue that exposure to a more conflictive family environment may demand more cognitive efforts from children, such as in studies with adolescents, where increased FC between PCC-mPFC is found in higher conflictive and lower cohesive families [42]. In this regard, our results support previous findings of stressor effects on the the frontolimbic circuitry (including the mPFC), which often describe increased FC in limbic regions [43,56] and in other DMN regions associated with interparental conflict [41]. Similarly, we found that increased FC on PCC-lIPL regions occurred when children were exposed to a lower cohesive family environment. Curiously, when the stressful experience becomes a trauma, similar results are described in adults: an increased FC in rIPL and PCC/precuneus in women who suffered sexual abuse in childhood [57], as well as enhanced regional homogeneity between lIPL and right superior frontal gyrus in men who survive earthquakes [58]. In both situations, the research participants were patients with post-traumatic stress disorder. Since our participants are not suffering from any mental illness and they are not exposed to extreme stress experiences, one possible hypothesis is that they are managing their stress situations with effective coping.

Effective coping means the ability to appraise the situation and choose an appropriate coping strategy as a response [59], and the DMN plays a role in this ability (i.e., adults with proactive coping) [60]. Thus, we conjecture that the more extensive IPL FC with the PCC and MPFC as a DMN cause a compensatory involvement and help individuals cope with stressful experiences. This is probably due to the influence of maternal behavior, once the increased FC of PCC with lIPL is moderated by more cohesive family functioning associated with a responsive behavior (e.g., maternal responsiveness). Findings from Che et al. [61] support our findings, they describe that the increased intra-DMN FC was associated with higher levels of perceived social support (in adults), interpreted as a key psychological factor in stress responses (i.e., adequate coping). In this case, we speculate that the maternal behavior might be acting to reduce the aversive response of family stress due to the emotional closeness to their children. We hypothesize that mothers act as their own reference for the socialization process and model their perspective-taking through their interactions with their children once the recruitment of IPL (on both PCC and mPFC regions) is thought to allow the distinction between self-generated actions and actions generated by others [62]. Interestingly, cohesive familiar functioning alone is associated with higher FC in both mPFC-lIPL and PCC-lIPL node regions. On the contrary, lower FC in both nodes is observed when maternal behavior mediates this association. Our findings are in line with Degeilh et al. [5], who demonstrated that effective maternal parenting is associated with DMN functioning, which in other words, means that early life parenting could shape the function of the developing brain [3].

Importantly, we need to consider that our study has important limitations that should be taken into account. First, the shift in how associations influence brain FC suggests that the effects of parenting may not be static but are likely to change across development [63]. To better understand this variation over time, longitudinal studies would be necessary, including repeated measurements over a long period of follow-up [64]. A second limitation is the use of the mothers’ self-report and not the examination of the children’s behavior. Additionally, family environment measures such as maternal behavior were assessed via a short maternal report. Moreover, our sample had a very small number of fathers (and we chose to exclude them). Increasing the number of parents in future studies (and other secondary caregivers) may also amplify the marginal findings obtained in this study. However, our findings are not explained by such restrictions. In future studies, the inclusion of observational measures will be important to assess these constructs. For an accurate assessment of the impact of the family environment quality on intrinsic DMN FC development, further studies might include a longitudinal approach and larger samples. Including genetic investigations will also be useful to clarify the effect of heritability from parents on a child’s brain and behaviors (as potential interpretation bias).

## 5. Conclusions

Our findings provide evidence that variations in the quality of the environment are associated with differences in the intrinsic DMN FC. We speculate that parental practices might improve children’s self-referential processes attributed to DMN functioning. Further studies will help to elucidate these gaps and test if improving relationships between the parent and the child might offer better support for neurodevelopmental processes and the regulation of socio-emotional abilities in children.

## Figures and Tables

**Table 1:** Summary statistics of key variables.

Variable	Minimum	1st Quartile	Median	Mean	Standard Deviation	3rd Quartile	Maximum	Missing	Occurrences
Age	6	7	9.00	8.68	1.79	10	12	0	-
FES Cohesion	1	5	6.00	5.69	1.19	6	9	0	-
FES Conflict	1	3	4.00	04.02	1.28	5	8	0	-
FES Total	1	4	5.00	4.76	1.42	6	9	0	-
SES	6	5	20.00	20.17	4.50	6	39	0	-
N total	**615**								

FES—family environment scale, SES—socioeconomic status.

**Table 2:** GLM coefficients for each DMN link, interpersonal family function (FES) and maternal parenting.

Independent Variables		beta (*p*-Value *; Standard Error; *t*-Value)	
Pair of Regions	Coefficient	Conflict	Cohesion
mPFC-PCC	**FES** **Parenting** FES * Parenting	0.197 (0.633; 0.632; 0.312) 0.612 (0.532; 0.979; 0.625) −1.053 (0.420; 1.304; −0.807)	−0.494 (0.466; 0.677; −0.729) 2.969 (0.597; 5.620; 0.528) −0.496 (0.610;.972;−0.510)
mPFC-rIPL		0.842 (0.156; 0.593; 1.421) −0.299 (0.644; 0.646; −0.462) −0.413 (0.651; 0.913; −0.452)	0.860 (0.153; 0.602; 1.429) 0.756 (0.873; 4.719; 0.160) −0.123 (0.920; 0.123;−0.101)
mPFC-lIPL		**1.344 (0.029; 0.617; 2.179)** 1.368 (0.804; 5.509; 0.248) −0.192 (0.840; 0.953; −0.201)	−0.181 (0.605; 0.664;−0.272) 0.670 (0.892; 4.912; 0.136) −0.094 (0.941; 1.273; −0.074)
PCC-lIPL		0.047 (0.962; 0.994; 0.047) 5.050 (0.517; 7.797; 0.648) −1.138 (0.574; 2.022; −0.563)	1.001 (0.344; 1.056; 0.948) **−11.485** (**0.046**; 5.748; −1.998) **2.323** (**0.020**; 1.002; 2.317)
PCC-rIPL		−0.302 (0.724; 0.856; −0.353) 2.998 (0.668; 2.993;−1.061) −0.465 (0.3504; 1.210; −0.384)	1.002 (0.209; 0.796; 1.258) 5.490 (0.380; 6.248; 0.879) −1.344 (0.407; 1.620;−0.830)

mPFC—medial prefrontal cortex; PCC—posterior cingulate cortex; rlPL—right lateral parietal cortex; llPL—left lateral parietal cortex; FES—family scale environment; * *p*-values uncorrected.

## Data Availability

Data are available under request.
